# Reconsideration of *Anopheles rivulorum* as a vector of *Plasmodium falciparum* in western Kenya: some evidence from biting time, blood preference, sporozoite positive rate, and pyrethroid resistance

**DOI:** 10.1186/1756-3305-5-230

**Published:** 2012-10-10

**Authors:** Hitoshi Kawada, Gabriel O Dida, George Sonye, Sammy M Njenga, Charles Mwandawiro, Noboru Minakawa

**Affiliations:** 1 Department of Vector Ecology & Environment, Institute of Tropical Medicine, Nagasaki University, Nagasaki, Japan; 2 School of Public Health, Maseno University, Kisumu, Kenya; 3 Springs of Hope, Mbita, Kenya; 4 Eastern and Southern Africa Centre of International Parasite Control, Nairobi, Kenya; 5 Kenya Medical Research Institute, Nairobi, Kenya; 6 The Global Center of Excellence Program, Nagasaki University, Nagasaki, Japan

**Keywords:** Permethrin, Deltamethrin, Resistance, *Anopheles rivulorum*, *Anopheles funestus*, *Plasmodium falciparum*, Kenya

## Abstract

**Background:**

*Anopheles gambiae*, *An. arabiensis*, and *An. funestus* are widespread malaria vectors in Africa. *Anopheles rivulorum* is the next most widespread species in the *An. funestus* group. The role of *An. rivulorum* as a malaria vector has not been fully studied, although it has been found to be a minor or opportunistic transmitter of *Plasmodium falciparum*.

**Methods:**

Mosquitoes were collected indoors over a 12-hour period using a light source attached to a rotating bottle collector in order to determine peak activity times and to provide DNA for meal source identification. Gravid female mosquitoes were collected indoors via an aspirator to generate F1 progeny for testing insecticidal susceptibility. Blood meal sources were identified using a multiplexed PCR assay for human and bovine cytochrome-B, and by matching sequences generated with primers targeting vertebrate and mammalian cytochrome-B segments to the Genbank database.

**Results:**

*Anopheles rivulorum* fed on human blood in the early evening between 18:00 and 20:00, when insecticide-treated bed nets are not in use, and the presence of *Plasmodium falciparum* sporozoites in 0.70% of the *An. rivulorum* individuals tested was demonstrated. Susceptibility to permethrin, deltamethrin, and DDT is higher in *An. rivulorum* (84.8%, 91.4%, and 100%, respectively) than in *An. funestus* s.s. (36.8%, 36.4%, and 70%, respectively), whereas mortality rates for propoxur and fenitrothion were 100% for both species. Resistance to pyrethroids was very high in *An. funestus* s.s. and the potential of the development of high resistance was suspected in *An. rivulorum*.

**Conclusion:**

Given the tendency for *An. rivulorum* to be active early in the evening, the presence of *P. falciparum* in the species, and the potential for the development of pyrethroid resistance, we strongly advocate reconsideration of the latent ability of this species as an epidemiologically important malaria vector.

## Background

*Anopheles gambiae* Giles, *An. arabiensis* Patton, and *An. funestus* Giles are widespread malaria vectors in Africa. Both *An. gambiae* s.s. and *An. funestus* s.s. are highly anthropophilic [1,2], while *An. arabiensis* prefers to feed on humans but will feed on other hosts as well [3]. A fourth species, *An. rivulorum* Leeson, is the next most widespread species in the *An. funestus* group [4]. The role of *An. rivulorum* as a malaria vector has not been fully studied [5-10]. Previous studies have demonstrated that *An. rivulorum* has the potential to replace other major malarial vectors such as *An. funestus* s.s. after spraying of indoor insecticides eliminates the more abundant species [11].

The use of insecticide-treated bed nets (ITNs) is a simple and inexpensive self-protection measure against malaria, which has been shown to reduce the morbidity of children (<5 years old) by 50% and global child mortality by 20%–30% [12,13]. The dramatic reduction in malaria transmission in Kenya [13] may be due to fewer opportunities for the major anthropophilic and endophagous vectors to feed successfully on humans. However, the increase in the use of ITNs in Kenya has led to an increase in insecticide resistance [14-16] and in the replacement of vector species [17].

This study reports the vectorial and behavioral characteristics and the insecticide susceptibility of *An. rivulorum* in western Kenya, and strongly recommends a reevaluation of this species as a minor vector of *P. falciparum*. A large portion of the paper reports on insecticide susceptibility as well.

## Methods

### Study area

The Gembe East area in the Mbita District of Nyanza Province in western Kenya was used as the study area. The area is drier in the eastern part of the district, and becomes progressively wetter towards the higher altitudes in the western parts of Gwassi Hills and Mfangano Island. In the highlands, the rainfall ranges between 800–1900 mm per annum, while the lowlands receive slightly less at 800–1200 mm each year. The rainfall pattern in the area is bimodal, with the long rainy season occurring from March through May, and the short rainy season occurring in November and December. Malaria infection rates rise steadily between September and February and peak briefly in June, following the long rains [18]. The Mbita and Suba districts are 2 focal points identified as high vector transmission areas in Kenya, and more than 50% of the population is exposed to malaria at a rate of ≥40% *Pf*PR_2-10_ (*Pf* parasite rate corrected to a standard age-range of 2 to less than 10 years old) [19]. Efforts have recently been renewed in this area to increase the use of effective preventative measures such as ITNs and combined vector control approaches. The Akado Medical Centre Project Mosquito Net, the Power of Love Foundation in partnership with World Swim Against Malaria, and the Ministry of Health distributed 6000 ITNs to children under 5 years of age and to pregnant women in the Gembe area. This increased the ITN coverage rate by at least 35%, from 17% to 52%.

### Adult mosquito collection and rearing of F1 progenies

Indoor adult collections took place in Nyandago, Nyaroya, Akuot, Alala, Alero, Kirindo, Ngou, and Uwi village in the Gembe East area on the shores of Lake Victoria. The house residents were informed about the study and their written consent was obtained before mosquito collection. Collections were performed during February 5–11, 2010; April 19–June 10, 2010; September 13–October 28, 2010; and January 27–February 4, 2012. Collections were done with a battery-powered aspirator (C-cell Aspirator, BioQuip Products, Rancho Dominguez, CA, USA) between 07:00–09:00 by 3 persons. After collection, gravid females were individually confined into a 20 ml glass vial containing 2 ml of water. A strip of filter paper (ca 3 × 4 cm) was placed inside each vial to collect eggs. Hatched larvae were reared with dechlorinated tap water until adult emergence. Larvae were fed a mixture of powdered animal food (CE-2; Clea Inc., Tokyo, Japan.) and dried yeast (Ebios®; Mitsubishi Tanabe Pharma, Tokyo, Japan). Grass leaves were added to rearing water containing *An. funestus* s.s. and *An. rivulorum* larvae to provide a resting place. The containers were exposed to sunlight to facilitate phytoplankton growth as a larval food resource [20]. Unfed F1 female adults were used for insecticide susceptibility tests.

### Adult mosquito collection by miniature light traps equipped with collection bottle rotators

Indoor mosquito trapping was done with 6 sets of the Center for Disease Control (CDC) miniature light trap (Model 512) equipped with a collection bottle rotator (Model 1512) (John W. Hock Co., Gainesville, FL, USA) in 3 houses in Nyaroya village and 4 houses in Nyandago village from September 16–October 5, 2010 and July 11–18, 2012. The house residents were informed about the study and their written consent was obtained before mosquito collection. The collection bottle rotator, which has 8 separate plastic collection bottles, was programmed to collect active mosquitoes at 2-hour intervals between 16:00–08:00. The traps were placed in the corner of the living room as far apart from the places where people sleep as possible. Female mosquitoes were identified and classified as unfed, blood-fed, and gravid. The abdominal contents of fed females were used for DNA extractions in order to identify the blood source.

### Identification of meal sources in blood-fed mosquitoes

Mosquitoes collected in the field were individually placed in 1.5 ml plastic tubes containing silica gel desiccant and stored at −10°C until processed. Engorged abdomens were separated from the rest of the body for blood source identification.

DNA was extracted from the blood in each abdomen using the REDExtract-N-Amp™ Tissue PCR Kit (SIGMA, St. Louis, MO, USA) per the manufacturer’s instructions. Species identification of the host blood source was performed by 2 different methods; multiplex PCR as described by Kent and Norris [21], or direct sequencing as described by Sawabe *et al.*[22].

Multiplex PCR was carried out using published cytochrome-B primers for human (Human741F, ggcttacttctcttcattctctcct) and cow (Cow121F, catcggcacaaatttagtcg) and a universal reverse primer (UNREV1025, ggttgtcctccaattcatgtta) [21]. A 20 μl cocktail consisting of 0.5 μM of each primer and previously diluted (300 times) 1 μl of the DNA template was placed in a 0.2 mL cell containing lyophilized AccuPower™ PCR Premix (BIONEER, Daejeon, Korea). The PCR was performed under the following conditions: a hot start at 95°C for 5 min; 35 cycles of template denaturing at 95°C for 1 min, primer annealing at 54°C for 1 min, and amplicon extension at 72°C for 1 min; and a final extension at 72°C for 7 min. Human and cow blood was detected by agarose gel electrophoresis (2% TAE) as 334 bp and 561 bp bands, respectively [21].

PCR amplification to generate amplicons for sequencing was done using published primers VerU-1 (aagacgagaagacccyatgga) and VerU-2 (cctgatccaacatmgaggtcgta) designed to detect the cytochrome-B gene of vertebrates; and Mammalian-1 (tgayatgaaaaaycatcg) and Mammalian-2 (tgtagttrtcwgggtckccta) cytochrome-B designed to detect the cytochrome-B gene of mammals [22]. The PCR mixture contained 4 μl of REDExtract-N-Amp™ ReadyMix cytochrome-B (SIGMA, St. Louis, MO, USA), 0.5 μM of each primer, and 1 μl of the DNA template in a total volume of 10 μl. PCR was conducted under the following conditions: a hot start of 94°C for 2 min; 35 cycles of template denaturing at 94°C for 30 sec, primer annealing at 55°C for 30 sec, and amplicon extension at 72°C for 1 min; and a final extension at 72°C for 4 min. Direct sequencing was performed with a 3730 DNA Analyzer (Applied Biosystems, Carlsbad, CA, USA). The results were analyzed by MEGA 4.0 public domain software ( http://www.megasoftware.net/). Sequences were used to perform basic local alignment searches against Genbank (National Center for Biotechnology Information website http://ww.ncbi.nlm.nih.gov/BLAST/) to determine the identities of the host species [22].

### Detection of *P. falciparum* in mosquitoes

Evidence of the presence of *P. falciparum* was first determined by enzyme-linked immunosorbent assay (ELISA). The head and thorax of each mosquito was homogenized in phosphate buffered saline (pH 7.4) and tested for the circumsporozoite antigen using a monoclonal antibody ELISA [23]. ELISA positive samples were confirmed by a *Plasmodium specific* PCR. DNA extraction was performed on the QIAcube (kit AIAamp DNA Micro Kit 56304, Qiagen, Tkyo, Japan) following the manufacturer’s instructions and the methods by Durnez *et al.*[24]. A 20 μl aliquot of the ELISA-lysate was added to 80 μl of ATL-buffer and extracted in the QIAcube machine, which eluted the DNA in 50 μl AE-buffer. The pestles used in the extraction had been sterilized in HCl solution to prevent contamination. The nested PCR was carried out according to the methods by Snounou *et al.*[25]. The first amplification was done using published primers rPLU-5 (cctgttgttgccttaaacttc) and rPLU-6 (ttaaaattgttgcagttaaaacg). The PCR mixture contained 0.4 μl of Mighty Amp DNA Polymerase (Takara Bio, Inc., Tokyo, Japan), 0.6 μM of each primer, and 1 μl of the DNA template in a total volume of 20 μl. PCR was conducted under the following conditions: a hot start of 98°C for 2 min; 35 cycles of template denaturing at 98°C for 10 sec, primer annealing at 58°C for 15 sec, and amplicon extension at 68°C for 1 min; and a final annealing at 57°C for 2 min and extension at 72°C for 4 min. The 2nd amplification was done using *Plasmodium falciparum* specific primers rVIV1 (cgcttctagcttaatccacataactgatac) and rVIV2 (acttccaagccgaagcaaagaaagtcctta). The PCR mixture contained 0.2 μl of Mighty Amp DNA Polymerase (Takara Bio, Inc., Tokyo, Japan), 0.3 μM of each primer, and 1 μl of the DNA template in a total volume of 10 μl. PCR was conducted under the following conditions: a hot start of 98°C for 2 min; 35 cycles of template denaturing at 98°C for 10 sec, primer annealing at 58°C for 15 sec, and amplicon extension at 68°C for 30 sec; and a final annealing at 57°C for 2 min and extension at 72°C for 4 min.

### Insecticide susceptibility tests using World Health Organization test tubes for F1 progenies

Adult susceptibility tests to insecticides was done using World Health Organization (WHO) test tube kits for F1 progenies and performed according to WHO instructions (WHO/CDS/CPC/MAL/98.12). Papers impregnated with either 0.75% permethrin, 0.05% deltamethrin, 4% DDT, 1% fenitrothion, or 0.1% propoxur were used for the tests. F1 larvae from the separate egg batches oviposited by field collected females were pooled in one batch to get adult females. Ten F1 1–3-day-old female mosquitoes were released into WHO test tubes for exposure to insecticide-impregnated paper for one hour, and the time to knockdown was recorded. One hr exposure to fenitrothion was performed in our bioassay, although 2 hr is stipulated in WHO instructions. Insects were then transferred to a clean tube, fed via cotton soaked with a 5% glucose solution, and mortality was recorded after one day. KT_50_ (time required for 50% knockdown) was obtained and average mortality was calculated. Two to 4 replications were performed for each insecticide. Control test was performed in each replication.

### Species identification

Mosquito adults were examined microscopically to distinguish *An. funestus* s.l. from other anophelines based on identification keys developed by Gillies and Coetzee [26]. Individual species within *An. funestus* s.l. were identified using the multiplex polymerase chain reaction (PCR) method described by Koekemoer *et al.*[27].

### Data analysis

KT_50_ was calculated by Bliss' probit method [28]. Mosquito densities were calculated as the mean number of female mosquitoes collected per collection time (2 hours) per house. The data for each replication were excluded from analysis when the total number of mosquitoes collected during a night was less than 5.

## Results

### Species abundance, *P. falciparum* sporozoite rate and blood meal composition

The total number of *An. funestus* s.l. female mosquitoes collected in the Gembe East area is shown in Table 1. The number of *An. funestus* s.s. was higher than *An. rivulorum* throughout the year in 2010. In contrast, the species composition was reversed in 2012.

**Table 1:** **Species composition of *An. funestus *s.l. collected in the Gembe East area in western Kenya**

**Species**	**Collection date - No. of females collected**				
	**2010**			**2012**	
	**Feb–Mar**	**Apr–Jul**	**Sept–Nov**	**Jan–Mar**	**Jul**
*An. funestus* s.s.	74	686	732	21	56
*An. rivulorum*	0	58	373	235	107
(*An. rivulorum* %)	(0.0)	(7.8)	(33.8)	(91.8)	(65.6)
*An. gambiae s.s.*	1	8	1	NA^1)^	NA
*An. arabiensis*	31	67	33	NA	NA

^1)^ NA: Collection of *An. gambiae* group was not done.

Of the 250 *An. funestus* s.s. and 284 *An. rivulorum* female mosquitoes used, 5 (2.0%) and 5 (1.76%) females were found to be *P. falciparum* positive by CSP-ELISA and 2 (0.80%) and 2 (0.70%) females were *P. falciparum* positive by PCR detection, respectively (Table 2).

**Table 2:** ***Plasmodium falciparum *sporozoite rate in *An. funestus *s.l. collected in the Gembe East area in western Kenya, Sept–Oct 2010**

**Species**	**No. of females used**	**No. of* P. falciparum *positive (ELISA**)	**No**. **of* P****.****falciparum *positive** (**PCR**)	**% positive**
*An. funestus* s.s.	250	5	2	0.80
*An. rivulorum*	284	5	2	0.70

Blood meal compositions for the 2 species are shown in Table 3. Proportion of human blood was higher in *An. funestus* s.s. (48.0%–50.7%) as compared to that in *An. rivulorum* (6.7%–16.0%). *An. rivulorum* was found to feed on various animals such as cows, bats, birds, apes, mice, bushbuck, and hippopotami; in comparison, *An. funestus* s.s. fed on cows, bats, and dogs.

**Table 3:** **Blood meal composition in female *An. funestus *s.l. collected in the Gembe East area in western Kenya**

	**Detection method**	**No**. **examined**	**Human blood** (**%**)	**Cow blood** (**%**)	**Others**	
					**No**. (**%**)	**Species**
*An. funestus* s.s.	Direct sequencing	25^a^	12 (48.0)	16 (64.0)	5 (20.0)	bats, dogs
	PCR	69^b^	35 (50.7)	4 (5.8)	32 (46.4)	-
*An. rivulorum*	Direct sequencing	148^a^	24 (16.0)	145 (98.0)	24 (16.0)	bats, birds, apes, mice, bushbuck, hippopotami
	PCR	89^c^	6 (6.7)	33 (37.1)	53 (59.6)	-

^a)^ collected in Oct 2011.

^b)^ collected in Feb 2011and Feb 2012.

^c)^ collected in Jan 2012.

### Indoor flight activity pattern of *An. rivulorum* females trapped by CDC miniature traps equipped with a collection bottle rotator

The houses used for indoor mosquito collections were of standard construction for the area, with mud walls and eaves, traditional opening structures between the roof and the walls, and divided into 1–2 bedrooms and 1 living room. The number of ITNs used in each household ranged from 0–2 (average 0.8 net/house), with 0–3 people sleeping in each bedroom (average 2.0 persons/bed room), and 0–4 people (mainly children > 5 years old) sleeping in each living room without ITNs (average 1.8 persons/living room). The number of cows, sheep, and goats reared around each house ranged from 0–17 (average 12.2), 0–8 (average 4.4), and 0–9 (average 6.0), respectively.

Among the 31 collections conducted, 13 were not used due to <5 mosquitoes collected on that night, and 4 collections had to be discarded due to equipment malfunction. The remaining 14 collections were used for analysis.

**Figure 1:**
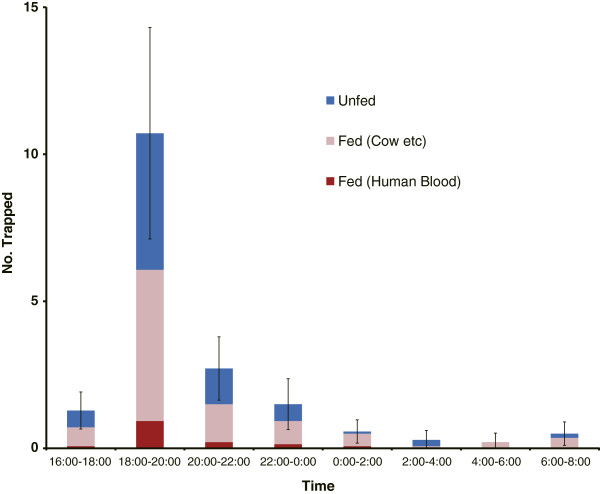
**Indoor activity pattern of *An. rivulorum *females.** Indoor activity pattern of *An. rivulorum* females trapped by Center for Disease Control miniature traps equipped with a collection bottle rotator. Bars indicate 95% confidential limits in total number of mosquitoes collected in each collection times.

The number of trapped *An. rivulorum* females rose sharply during early evening (18:00–20:00), falling drastically after this period (Figure 1). The number of blood-fed females also peaked in this period. Among the 249 females collected, 104 were unfed (41.8%), 20 contained blood from a human source (8.1%), and 125 had fed from an animal source (50.2%). Among the 20 females engorged with human blood, 13 (65%) were trapped in the 18:00–20:00 period.

### Insecticidal susceptibility of *An. funestus* s.l. female adults

**Figure 2:**
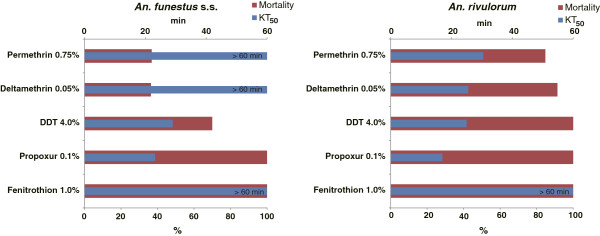
**Insecticide susceptibility of *An. funestus *F1 females.** Insecticide susceptibility of unfed female F1 adult mosquitoes in *An. funestus* s.l. determined by the World Health Organization tube test.

Insecticide susceptibility of adult F1 *An. funestus* s.l. female mosquitoes is shown in Figure 2.

*An. funestus* s.s. showed high resistance to both permethrin and deltamethrin (36.8% and 36.4% mortality, both >60 min KT_50_, respectively), while the resistance against DDT was lower (70.0% mortality and 29 min KT_50_). KT_50_ and mortality for propoxur and fenitrothion were 23.2 min - 100%, and >60 min - 100%, indicating that *An. funestus* s.s. is susceptible to these insecticides. Susceptibility of *An. rivulorum* to pyrethroids was higher compared to *An. funestus* s.s. but resistance was suspected in this species according to WHO criteria, since mortalities to permethrin and deltamethrin were less than 100% (84.8% and 91.4%, respectively), while KT_50_s were smaller than those in *An. funestus* s.s. (30.5 min and 25.5 min, respectively). Susceptibility of *An. rivulorum* against DDT, propoxur, and fenitrothion was high (25 min KT_50_ - 100% mortality, 17 min KT_50_ - 100% mortality, and >60 min KT_50_ - 100% mortality, respectively).

## Discussion

The importance of *An. rivulorum* as a vector of human malaria in Africa was first advocated by Wilkes *et al.* in 1996 [29]. Most of the mosquito sampling in that study was done at resting sites close to cattle corrals and pit shelters. All-night landing catches on human bait were done outdoors, since this species was considered to be exophilic and zoophilic [4]. Some reports, however, presented evidence that this species was neither completely exophilic nor zoophilic. Oyewole and Awolola reported that 42% of *An. rivulorum* in Lagos, Nigeria was collected indoors [8]. All mosquito collections were performed indoors in the current study. The increase in the proportion of *An. rivulorum* from 2010 to 2012 possibly seemed to be due to a rise in the availability of larval habitats. Larvae of *An. rivulorum* are closely associated with aquatic vegetation, such as the water hyacinths in Lake Victoria, and the fluctuation of the lake water levels may have affected habitat availability [30].

*Plasmodium falciparum* in *An. rivulorum* was absent or low in previous reports: 0% (0/80) [7], 0% (0/90) [8], 0% (0/264) [10], and 0.5% (1/203) [29]. Wilkes *et al.* reported that 0.5% of *An. rivulorum* (5/1022) was positive via observation of sporozoites using a compound microscope, and that 1 of the 2 samples examined by ELISA was *P. falciparum* positive [29]. Temu *et al.* detected 2 *Pf* positive individuals in 10 *An. rivulorum* when examined by nested polymerase chain reaction (PCR), although the number of samples was not enough to be statistically evaluated [6]. The *P. falciparum* positive rate for *An. rivulorum* in the present study (0.70%) seemed to be relatively high compared to previous reports.

*Anopheles rivulorum* is more zoophilic than *An. funestus* s.s. and a broad range of animals were found to be serving as hosts. Host preference, however, appears to be affected by the human/animal ratio, as mosquitoes are more zoophilic in an area where animals are more abundant than humans [31]. A high level of anthropophily in *An. rivulorum* was reported by Kweka *et al.*[7], indicating that 64.5% of *An. rivulorum* sampled in north-eastern Tanzania had taken a blood meal from a human host. Awolola *et al.* reported that 39.6% of *An. rivulorum* were found to have ingested human blood in a forest area of southern Nigeria [10]. Likewise, Wilkes *et al.* collected a number of *An. rivulorum* using human bait (>14 females/h) [29]. The evidence above indicates that this species has the potential to be a transmitter of human malaria. In our study, a remarkable flight activity peak of *An. rivulorum* was observed in the early evening (18:00–20:00), indicating that this species prefers to feed during a period before humans are likely to be in bed and under an ITN. These data correlate with the results shown by Wilkes *et al.* using human bait [29].

The difference in pyrethroid susceptibilities between *An. funestus* s.s. and *An. rivulorum* may be explained by the different probabilities of each species coming into contact with the pyrethroids thought to be used exclusively in ITNs. *An. funestus* s.s. and 2 other major vectors in the study area, *An. gambiae* s.s. and *An. arabiensis*, have developed high pyrethroid resistance via unique mechanisms [14,15]. Pyrethroid resistance in *An. gambiae* s.s. has developed by the dramatic increase of the L1014S mutation in the voltage gated sodium channel (*kdr*), and mixed function oxydase-related metabolic resistance has developed in both *An. arabiensis* and *An. funestus* s.s. [14]. These resistances were thought to be caused solely by wide-spread distribution of ITNs containing pyrethroids in the study area during the past decade. Indoor spraying with residual insecticides has not been done in the study area [14]; therefore, neither carbamates nor organophosphates are supposed to have been used to control malaria vectors. However, the chance of *An. rivulorum* being exposed to the pyrethroids impregnated in most of the ITNs seems to be low compared to other vector species, as most of the feeding in this species takes place in early evening. Although most people may not be under the ITNs early in evening, the nets are still in houses hung or folded, making a chance of exposure for mosquitoes. The mortalities on permethrin and deltamethrin (<100%) indicate that the selection pressure of pyrethroids on this species is not negligible. The monitoring of pyrethroid resistance is therefore necessary in *An. rivulorum* as in the other vector species.

## Conclusion

In conclusion, the present report clarifies that *An. rivulorum* feeds on human blood, especially in early evening when ITNs are not in use. *Plasmodium falciparum* sporozoites were detected in 0.70% of mosquitoes tested, and presents data indicating a possibility that this species has developed pyrethroid resistance according to the latest WHO criteria (Global Plan for Insecticide Resistance Management in Malaria Vectors; http://www.who.int/malaria/vector_control/ivm/gpirm/en/index.html). Therefore, we strongly recommend that the latent ability of this species as a minor vector should be reconsidered and that the urgent need for implementation of a resistance management strategy for such species. The development of new technologies to control such malaria vectors will also be essential [32].

## Competing interests

The authors declare that they have no competing interests.

## Authors’ contributions

HK designed the study, carried out the experiments, and drafted the manuscript. GOD and GS arranged the fields and informed consents of the participants for the study and organized the staffs for the experiments. SMN, CM, and NM critically revised the protocol for the study. All authors read and approved the final version of the manuscript.

## References

[B1] Mbogo CN Kabiru EW Muiruri SK Nzovu JM Ouma JH Githure JI Beier JC Bloodfeeding behavior of *Anopheles gambiae* s.l. and *Anopheles funestus* in Kilifi District, Kenya J Am Mosq Control Assoc 1993 9 225 227 8350080

[B2] Githeko AK Service MW Mbogo CM Atieli FK Juma FO Origin of blood meals in indoor and outdoor resting malaria vectors in western Kenya Acta Trop 1994 58 307 316 10.1016/0001-706X(94)90024-8 7709869

[B3] Muriu SM Muturi EJ Shililu JI Mbogo CM Mwangangi JM Jacob BG Irungu LW Mukabana RW Githure JI Novak RJ Host choice and multiple blood feeding behaviour of malaria vectors and other anophelines in Mwea rice scheme Kenya. Malaria J 2008 7 43 10.1186/1475-2875-7-43 PMC2291060 18312667

[B4] Gillies MT De Meillon B Gillies MT De Meillon B The Anophelinae of Africa South of the Sahara Publications of the South African Institute for Medical Research 1968 Johannesburg Volume 54

[B5] Hargreaves K Koekemoer LL Brooke BD Hunt RH Mthembu J Coetzee M *Anopheles funestus* resistant to pyrethroid insecticides in South Africa Med Vet Entomol 2000 14 181 189 10.1046/j.1365-2915.2000.00234.x 10872862

[B6] Temu EA Minjas JN Tuno N Kawada H Takagi M Identification of four members of the *Anopheles funestus* (Diptera: Culicidae) group and their role in *Plasmodium falciparum* transmission in Bagamoyo coastal Tanzania Acta Trop 2007 102 119 125 10.1016/j.actatropica.2007.04.009 17537390

[B7] Kweka EJ Mahande AM Nkya WM Assenga C Lyatuu EE Nyale E Mosha FW Mwakalinga SB Temu EA Vector species composition and malaria infectivity rates in Mkuzi, Muheza District, north-eastern Tanzania Tanzan J Health Res 2008 10 46 49 18680965 10.4314/thrb.v10i1.14341

[B8] Oyewole IO Awolola TS Impact of urbanization on bionomics and distribution of malaria vectors in Lagos, southern Nigeria J Vect Borne Dis 2006 43 173 178 17175702

[B9] Awolola TS Ibrahim K Okorie T Koekemoer LL Hunt RH Coetzee M Species composition and biting activities of anthropophilic *Anopheles* mosquitoes and their role in malaria transmission in a holoendemic area of southern Nigeria African Entomol 2003 11 227 232

[B10] Awolola TS Oyewole IO Koekemoer LL Coetzee M Identification of three members of the *Anopheles funestus* (Diptera: Culicidae) group and their role in malaria transmission in two ecological zones in Nigeria Trans R Soc Trop Med Hyg 2005 99 525 531 10.1016/j.trstmh.2004.12.003 15869772

[B11] Gillies MT Smith A The effect of a residual house spraying campaign in East Africa on species balance in the *Anopheles funestus* group: the replacement of *Anopheles funestus* Giles by *Anopheles rivulorum* Leeson Bull Entomol Res 1960 51 243 252 10.1017/S0007485300057953

[B12] Binka FN Kubaje A Adjuik M Williams LA Lengeler C Maude GH Armah GE Kajihara B Adiamah JH Smith PG Impact of permethrin treated bednets in child mortality in Kassena-Nankana district, Ghana: a randomized controlled trial Trop Med Int Health 1996 1 147 154 8665378 10.1111/j.1365-3156.1996.tb00020.x

[B13] Nevill CG Some ES Mung'ala VO Mutemi W New L Marsh K Lengeler C Snow RW Insecticide-treated bed nets reduce mortality and severe morbidity from among children on the Kenyan Coast Trop Med Int Health 1996 1 139 146 8665377 10.1111/j.1365-3156.1996.tb00019.x

[B14] Kawada H Dida GO Ohashi K Komagata O Kasai S Tomita T Sonye G Maekawa Y Mwatele C Njenga SM Mwandawiro C Takagi M Multimodal pyrethroid resistance in malaria vectors, Anopheles gambiae s.s., Anopheles arabiensis, and Anopheles funestus s.s. in western Kenya PLoS One 2011 6 e22574 10.1371/journal.pone.0022574 21853038 PMC3154902

[B15] Kawada H Futami K Komagata O Kasai S Tomita T Sonye G Mwatele C Njenga SM Mwandawiro C Minakawa N Takagi M Distribution of a knockdown resistance mutation (L1014S) in Anopheles gambiae s.s. and Anopheles arabiensis in Western and Southern Kenya PLoS One 2011 6 e24323 10.1371/journal.pone.0024323 21931682 PMC3170322

[B16] Mathias D Ochomo EO Atieli F Ombok M Bayoh MN Olang G Muhia D Kamau L Vulule JM Hamel MJ Hawley WA Gimnig JE Spatial and temporal variation in the kdr allele L1014S in Anopheles gambiae s.s. and phenotypic variability in susceptibility to insecticides in Western Kenya Malaria J 2011 10 10 10.1186/1475-2875-10-10 PMC3029224 21235783

[B17] Bayoh MN Mathias DK Odiere MR Mutuku FM Kamau L Gimnig JE Vulule JM Hawley WA Hamel MJ Walker ED *Anopheles gambiae*: historical population decline associated with regional distribution of insecticide-treated bed nets in western Nyanza Province Kenya Malaria J 2010 9 62 10.1186/1475-2875-9-62 PMC2838909 20187956

[B18] Gouagna LC Okech BA Kabiru EW Killeen GF Obare P Ombonya S Bier JC Knols BG Githure JI Yan G Infectivity of *Plasmodium falciparum* gametocytes in patients attending rural health centers in western Kenya East Afr Med J 2003 80 627 634 15018419 10.4314/eamj.v80i12.8779

[B19] Noor AM Patil AP Hay SI Muchiri E Patil AP Hay SI Muchiri E Muchiri E Muchiri E Alegana1 VA The risks of malaria infection in Kenya in 2009 BMC Infect Dis 2009 9 180 10.1186/1471-2334-9-180 19930552 PMC2783030

[B20] Tuno N Githeko AK Nakayama T Minakawa N Takagi M Yan G The association between the phytoplankton, *Rhopalosolen* species (Chlorophyta; Chlorophyceae), and *Anopheles gambiae* sensu lato (Diptera: Culicidae) larval abundance in western Kenya Ecol Res 2006 21 476 482 10.1007/s11284-005-0131-0

[B21] Kent BJ Norris DE Identification of mammalian blood meals in mosquitoes by a multiplexed polymerase chain reaction targeting cytochrome B Am J Trop Med Hyg 2005 73 336 342 16103600 PMC4147110

[B22] Sawabe K Isawa H Hoshino K Sasaki T Roychoudhury S Higa Y Kasai S Tsuda Y Nishiumi I Hisai N Hamao S Kobayashi M Host-feeding habits of *Culex pipiens* and *Aedes albopictus* (Diptera: Culicidae) collected at the urban and suburban residential areas of Japan J Med Entomol 2010 47 442 450 10.1603/ME09256 20496592

[B23] Wirtz RA Zavala F Charoenvit Y Campbell GH Burkot TR Schneider I Esser KM Beaudoin RL Andre RG Comparative testing of monoclonal antibodies against *Plasmodium falciparum* sporozoites for ELISA development Bull WHO 1987 65 39 45 3555879 PMC2490858

[B24] Durnez L Van Bortel W Denis L Roelants P Veracx A Trung HD Sochantha T Coosemans M False positive circumsporozoite protein ELISA: a challenge for the estimation of the entomological inoculation rate of malaria and for vector incrimination Malaria J 2011 10 195 10.1186/1475-2875-10-195 PMC3160429 21767376

[B25] Snounou G Viriyakosol S Zhu XP Jarra W Pinheiro L Do Rosario VE Thaithong S Brown KN High sensitivity of detection of human malaria parasites by the use of nested polymerase chain reaction Mol Biochem Parasitol 1993 61 315 320 10.1016/0166-6851(93)90077-B 8264734

[B26] Gillies MT Coetzee M A supplement to the Anophelinae of Africa south of the Sahara (Afrotropical region) South African Institute for Medical Research 1987 Volume 55

[B27] Koekemoer LL Kamau L Hunt RH Coetzee M A cocktail polymerase chain reaction (PCR) assay to identify members of the *Anopheles funestus* (Diptera: Culicidae) group Am J Trop Med Hyg 2002 66 804 811 12224596 10.4269/ajtmh.2002.66.804

[B28] Bliss CI The method of probits - A correction Science 1934 79 409 410 10.1126/science.79.2053.409 17792220

[B29] Wilkes TJ Matola YG Charlwood JD *Anopheles rivulorum*, a vector of human malaria in Africa Med Vet Entomol 1996 10 108 110 10.1111/j.1365-2915.1996.tb00092.x 8834753

[B30] Minakawa N Dida GO Sonye GO Futami K Njenga SM Malaria vectors in Lake Victoria and adjacent habitats in western Kenya PLoS One 2012 7 e32725 10.1371/journal.pone.0032725 22412913 PMC3297610

[B31] Tirados I Costantini C Gibson G Torr SJ Blood-feeding behaviour of the malarial mosquito *Anopheles arabiensis*: implications for vector control Med Vet Entomol 2006 20 425 437 10.1111/j.1365-2915.2006.652.x 17199754

[B32] Kawada H Dida GO Ohashi K Sonye G Njenga SM Mwandawiro C Minakawa N Takagi M Preliminary evaluation of the insecticide-impregnated ceiling nets with coarse mesh size as a barrier against the invasion of malaria vectors Jpn J Infect Dis 2012 65 243 246 22627307 10.7883/yoken.65.243

